# Does Renal Sympathetic Denervation Impact the Outcome in Sepsis-Induced Shock? An Experimental Porcine Model

**DOI:** 10.3390/jcm15134983

**Published:** 2026-06-26

**Authors:** Aikaterini Bratko, Apostolos Kamparoudis, Michael Doumas, Konstantinos Ballas, Stergios Arapoglou, Iliana Tzortzi, Ioannis Savvas, Christina Marouda, Dimitra Psalla, Georgios Zacharioudakis

**Affiliations:** 1 Fifth Department of Surgery, “Hippokration” General Hospital of Thessaloniki, Aristotle University of Thessaloniki, 54642 Thessaloniki, Greece; kambarou@med.auth.gr (A.K.); mpallas@auth.gr (K.B.); arapos1@hotmail.com (S.A.); zachsgr@auth.gr (G.Z.); 2 Second Propedeutic Department of Internal Medicine, “Hippokration” General Hospital of Thessaloniki, Aristotle University of Thessaloniki, 54642 Thessaloniki, Greece; doumasm@auth.gr; 3 Companion Animal Clinic, School of Veterinary Medicine, Faculty of Health Sciences, Aristotle University of Thessaloniki, 54124 Thessaloniki, Greece; tzortzii16@gmail.com (I.T.); isavas@vet.auth.gr (I.S.); 4 Laboratory of Pathology, School of Veterinary Medicine, Faculty of Health Sciences, Aristotle University of Thessaloniki, 54124 Thessaloniki, Greece; cmarouda@vet.auth.gr (C.M.); dpsalla@vet.auth.gr (D.P.)

**Keywords:** renal sympathetic denervation, sepsis, septic shock, organ failure, inflammatory response

## Abstract

**Background/Objectives**: Septic shock remains a life-threatening condition characterized by systemic inflammation and hemodynamic instability leading to multiorgan dysfunction with a high mortality rate. The sympathetic nervous system (SNS) plays a central role in septic shock pathophysiology, while the impact of renal sympathetic denervation (RSD) remains unclear. **Methods**: In this study, 14 Large White pigs were randomized to bilateral RSD (R group, n = 9) or control (C group, n = 5). Septic shock was induced by causing fecal peritonitis. Hemodynamic parameters (systolic and diastolic blood pressure, heart rate), urine output, laboratory biomarkers (inflammatory, coagulation, renal, hepatic, and electrolytes), and organ histopathology were evaluated preoperatively, postoperatively, and 6 h after shock induction. **Results**: RSD induced a significant reduction in systolic and diastolic blood pressure along with an increase in heart rate and a decrease in urine output. During septic shock, the R group demonstrated greater prolongation of prothrombin time and international normalized ratio. Renal dysfunction, inflammatory markers, hepatic injury, and electrolyte disturbances showed no significant differences, similar to the histopathological findings. **Conclusions**: RSD alters cardiovascular and coagulation parameters without confirmed alterations in progression of renal dysfunction or systemic inflammatory and hepatic responses, indicating a role of the SNS during septic shock.

## 1. Introduction

Septic shock is a life-threatening condition marked by systemic inflammation and profound vascular dysregulation, resulting in multiorgan dysfunction and a high mortality rate [1,2]. Peritonitis due to abdominal infections—as in bowel perforation—is a recognized cause of sepsis and septic shock, as defined by the third international consensus definition (Sepsis-3) [2,3]. The pathophysiology of septic shock is multifaceted: several mechanisms may interact and lead to acute kidney injury in septic patients, including systemic and renal inflammation, microcirculatory and microcirculatory abnormalities, mitochondrial dysfunction and dysregulation of the renin–angiotensin–aldosterone system [4,5].

The sympathetic nervous system (SNS) plays a pivotal role in septic shock pathophysiology. The renal sympathetic nerves, distributed circumferentially around the renal arteries, regulate renal vascular tone, tubular sodium reabsorption, and renin release. However, during sepsis sympathetic hyperactivation may contribute to induction of organ failure. Experimental studies have demonstrated that excessive cytokine release is limited after inhibition of central sympathetic activation [6]. Recently, the central nervous system has been recognized as influencing the function of distant organs through the autonomic nervous system and the neuroimmune regulatory network [7,8]. This axis is suggested to play a central role in the systemic inflammatory pathway in septic shock, lending further weight to renal sympathetic modulation as a meaningful therapeutic target [9].

Renal sympathetic denervation (RSD) was introduced several decades ago as a therapeutic option in conditions such as resistant hypertension and other cardiovascular diseases [10,11,12,13]. Although the initial results were controversial, recent studies have confirmed the effectiveness and safety of RSD in controlling resistant hypertension [14,15,16,17,18,19,20]. Despite the well-established role of RSD in management of hypertension, its effects in the context of septic shock remain poorly understood. One could hypothesize that the interruption of renal sympathetic nerve activity may negatively affect the outcome of septic shock due to irreversible SNS inhibition. Only scarce experimental data are available: studies in rodent and ovine models have concluded in conflicting results as some presented the protective effects of RSD by attenuating inflammatory signaling [21,22] and others demonstrated deterioration of acute kidney injury [23]. However, none of the respective studies have been conducted in porcine models.

Obviously, ethical constraints limit the feasibility of conducting controlled studies on septic shock in humans; consequently, experimental animal models are used to gain translational insights. Porcine models are particularly appropriate due to their close similarity to humans in anatomy, physiology, genetics, and immune responses [24,25].

The present study hypothesized that bilateral RSD would affect the systemic inflammatory response and renal function in septic shock. A prospective, randomized, controlled experimental design was used, with renal function as the primary outcome and with hemodynamic, coagulation, inflammatory, and hepatic parameters, and, also, histopathology, as secondary outcomes in a porcine model of peritonitis-induced septic shock. Addressing this may offer preliminary mechanistic insight into the role of renal sympathetic signaling during septic shock, though direct extrapolation to patients with prior catheter-based RSD requires caution given the acute nature of the denervation model.

## 2. Materials and Methods

### 2.1. Animals and Preparation

The present study was approved by the Bioethical Committee of Aristotle University of Thessaloniki, the Ethics Committee of Companion Animal Clinic of School of Veterinary Medicine of Aristotle University of Thessaloniki (registration num:EL-54-BIOexp-18, protocol num: 85/2023, date of approval: 16 February 2023)) and the Animal Care Committee of the Region of Central Macedonia of Greece (protocol num: 889183(3911), date of approval: 14 December 2022, and protocol num: 54162(167)/9 February 2024).

The study included 14 Large White pigs: 10 male and 4 female, aged 12–16 months and with a body weight of 25–30 kg. All animals in the experimental protocol were laboratory animals, not privately owned, housed in specially designed stainless-steel cages for pigs, with free access to food and water and under controlled environmental conditions (temperature 18–22 °C, relative humidity 55–65%, and a 12 h light/dark cycle).

### 2.2. Anesthesia and Ventilation

All animals received pre-anesthetic medication consisting of midazolam (0.1 mg/kg, Richmond Vet Pharma Inc., Buenos Aires, Argentina), butorphanol (0.1 mg/kg, Vetviva Richter GmbH, Wels, Austria), medetomidine (0.1 mg/kg, Eurovet Animal Health, Bladel, Netherlands), and ketamine (10 mg/kg, Vetviva Richter GmbH, Wels, Austria), administered intramuscularly into the psoas muscles. After the pre-anesthetic medication, animals were subsequently transferred to the operating table of a fully equipped experimental operating theater at the Companion Animal Clinic. Catheterization of the auricular vein was performed using an Abbocath (22 G, Polymed Medicure Limited, New Delhi, India), to establish peripheral venous access. General anesthesia was induced with intravenous administration of propofol (2–3 mg/kg, CP-Pharma Handlesgesellschaft mbH, Burgdorf, Germany) followed by endotracheal intubation with a size 7–8 endotracheal tube (Jiangsu Hendhong Medical technology Co., Ltd., Yizheng, China). General anesthesia was maintained with administration of 100% oxygen and isoflurane (1–2%, Piramal Critical Care B.V. Inc., Voorschoten, The Netherlands) until the end of the study. Hemodynamic support was provided by intravenous administration of lactated Ringer’s solution (10 mL/kg/h, Bradex Inc., Krioneri, Greece). In addition, intravenous ampicillin (5 g, Intervet Hellas Inc., Athens, Greece) was administered as preoperative antimicrobial prophylaxis [26].

### 2.3. Experimental Protocol and Surgical Technique

**Figure 1:**
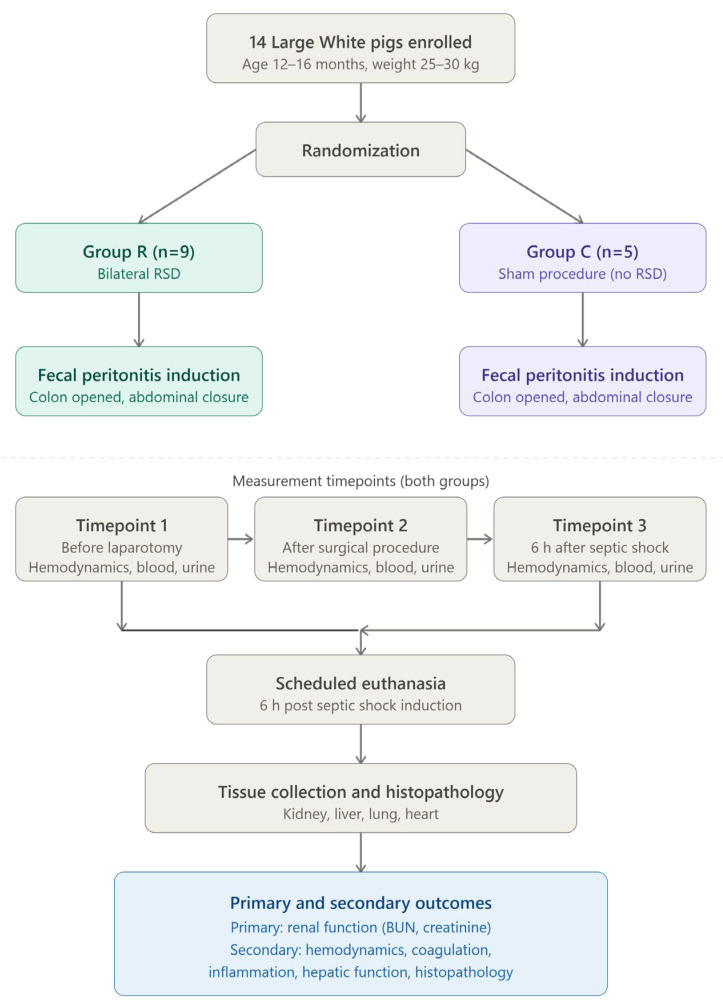
Study design flowchart. Fourteen Large White pigs were randomly allocated into two groups: group R (renal sympathetic denervation, n = 9) and group C (control, n = 5). Following group-specific surgical procedures and fecal peritonitis induction, hemodynamic parameters, urine output, and blood samples were collected at three timepoints: before laparotomy (T1), after the surgical procedure (T2), and six hours after septic shock induction (T3). Scheduled euthanasia was performed at six hours after septic shock induction, followed by immediate tissue collection for histopathological analysis. BUN: blood urea nitrogen; RSD: renal sympathetic denervation.

Laboratory animals were randomly allocated into two groups: (i) group R for pigs undergoing bilateral RSD of the renal vessels (n = 9) or (ii) group C for pigs undergoing the same procedure without RSD (n = 5) (Figure 1).

The abdominal surgical field was sterilized by using 10% povidone–iodine solution (Betadine, Lavipharm Inc., Athens, Greece) and applying sterile draping for sterilization. A midline laparotomy incision was performed to enter the peritoneal cavity, through the musculoaponeurotic layers of the abdominal wall. Bladder catheterization was performed with placement of a suprapubic urinary catheter (14Fr 2-way Foley, Shanghai International Holding Corp. GmbH, Hamburg, Germany) and transmural exteriorization through the abdominal wall with cutaneous fixation to the anterior abdominal wall. Subsequently, bilateral incision of the lateral peritoneal reflections of the parietal peritoneum was performed in order to mobilize the colon and allow access to the retroperitoneal space, the kidneys, and the renal vessels. In group C the procedure was completed after access to the retroperitoneal space and exposure of the renal vessels, without performing RSD. In group R, circumferential dissection and complete skeletonization of the renal arteries and their branches were performed bilaterally along their entire course, from their origin at the abdominal aorta to the renal hilum. Perivascular tissue was excised to ensure interruption of bilateral renal sympathetic neural signaling. In all animals, the colon was subsequently opened, and fecal material was released into the peritoneal cavity to induce fecal peritonitis and septic shock according to the criteria. Finally, the abdominal wall was closed with continuous sutures (PDS Plus suture 1, Ethicon J&J Med Tech, Edinburgh, Scotland), and the skin was closed with continuous sutures (Prolene suture 2/0, Ethicon J&J Med Tech, Edinburgh, Scotland).

During the first phase of the experimental protocol (surgical intervention), animals received intravenous paracetamol (20 mg/kg, Fresenius Kabi Hellas Inc., Athens, Greece) and intramuscular fentanyl (0.05 mg/kg, Kalcex Inc., Riga, Latvia) every 2 h. During the second phase (septic shock), additional analgesia was administered, including intramuscular morphine (3 mg/kg, Molteni Pharmaceutics Inc., Florence, Italy) and intravenous tramadol (2 mg/kg, Molteni Pharmaceutics Inc., Florence, Italy) for comfort reasons [26,27,28,29,30,31].

### 2.4. Autopsy and Histopathological Analysis

Scheduled euthanasia was performed 6 h after the induction of fecal peritonitis and septic shock by receiving an intravenous bolus overdose of propofol (20 mg/kg, CP-Pharma Handlesgesellschaft mbH, Burgdorf, Germany) and an intravenous bolus dose of potassium chloride (10 mL, DemoS.A., Athens, Greece), in accordance with the fundamental principles of laboratory animal euthanasia. Death was confirmed by cessation of spontaneous respiration and absence of cardiac activity on continuous electrocardiographic monitoring. Organ collection was initiated immediately following confirmation of death to minimize warm ischemia time and preserve tissue integrity for histopathological analysis. The previous abdominal wall incision was reopened to re-enter the abdominal cavity. The kidneys were excised en bloc with the renal vessels, and tissue samples from the liver were collected. Moreover, access to the thoracic cavity was achieved transdiaphragmatically following diaphragmatic incision for collection of tissue samples from lungs and heart. All samples were fixed in 10% neutral buffered formalin (Richard-Allan Scientific LLC, Thermo Fisher Scientific, Kalamazoo, MI, USA) and prepared for histological examination. Histological samples were prepared using standard paraffin-embedding techniques. Tissue sections of 4 μm thickness were stained with hematoxylin and eosin (H&E, Merck KGaA, Darmstadt, Germany) and evaluated microscopically. Each tissue section was examined under light microscopy for the presence or absence of pathological lesions. Kidneys were assessed for acute tubular injury, glomerular damage, interstitial capillary congestion and microhemorrhages. Liver samples were evaluated for capillary congestion, degenerative hepatocellular changes, periportal infiltration and Kupffer cell hyperplasia. Lung samples were examined for alveolar injury, edema, leukocytic infiltration, capillary congestion, atelectasis, and microhemorrhages. Heart samples were assessed for edema by measuring the interfibrillar distance, congestion increase in capillaries, microscopic bleeding and leukocytic infiltration. Histopathological analysis was performed by a pathologist blinded to group allocation. Laboratory analyses were processed without knowledge of group assignment.

### 2.5. Hemodynamics, Monitoring and Urine Output

During both phases of the experiment, the surgical procedure and septic shock, hemodynamic parameters were continuously monitored in both groups by using a non-invasive sphygmomanometer (Mindray Medical International Limited, Shenzhen, China) applied to the thigh of each animal, which provided intermittent measurements of systolic (SBP) and diastolic (DBP) arterial pressure, as well as heart rate (HR). Invasive arterial pressure monitoring, cardiac output measurement, and serum lactate quantification were not performed in this study, representing limitations in the characterization of shock severity. Septic shock was defined operationally by the combination of hypotension, tachycardia, decreased urine output, and biochemical evidence of organ dysfunction including rising BUN and creatinine, consistent with criteria applied in comparable porcine experimental sepsis models. Measurements were recorded prior to laparotomy in both groups. In group R, values were additionally recorded after RSD, whereas in group C measurements were taken after access to the retroperitoneal space. Finally, measurements were obtained after the induction of fecal peritonitis and septic shock in both groups. At the same timepoints, urine output (URINE) was assessed via Foley urinary catheters.

### 2.6. Laboratory Parameters

Each pig underwent a total of three blood samplings, i.e., prior to laparotomy, after the surgical procedure, 6 h after induction of septic shock, and before euthanasia. Approximately, a blood sample of 7 mL was collected from a central vein (femoral or jugular) using a syringe (21 G, 10 mL, Jiangsu Kanghua Medical Equipment Co., Ltd., Changzhou, China) and transferred into blood collection tubes (Golden Vac^TM^, Zhejiang Gongdong Medical Technology Co., Ltd., Taizhou, Zhejiang, China): 1.8 mL for the coagulation profile, 2 mL for full blood count and 3 mL for biochemical analysis, which were subsequently sent for laboratory analysis. The measured parameters included white blood cell count (WBC), platelet count (PLT) and coagulation parameters, specifically prothrombin time (PT), activated partial thromboplastin time (aPTT), and international normalized ratio (INR). Biochemical analysis was also performed, including measurement of blood urea nitrogen (BUN), creatinine (Creat), potassium (K), sodium (Na), aspartate aminotransferase (AST/SGOT) and alanine aminotransferase (ALT/SGPT), and C-reactive protein (CRP).

### 2.7. Statistical Analysis

A sample size estimation was performed prior to the study using G*Power 3.1.9.7, with a significance level of α = 0.05, statistical power of 80%, and an effect size of Cohen’s d = 1.5. This resulted in a total sample size of 14 animals (N = 9 in group R, and N = 5 in group C).

Data are presented as means ± SD. Statistical analysis was performed using R: A Language and Environment for Statistical Computing, version 4.5.1 (R Foundation for Statistical Computing, Vienna, Austria) with the Ime4, package version 2.0.1, ImerTest package version 3.2.0 and emmeans package version 2.0.3. Linear mixed-effects models were applied with group, time and their interaction as fixed effects, and subject-specific random intercepts to account for repeated measurements, with denominator degrees of freedom estimated using Satterthwaite’s method. Within-group and between-group contrasts at each timepoint were examined using estimated marginal means with the Holm adjustment for pair wise comparison. For qualitative variables, odds ratios (ORs) and expected frequencies (EFs) were calculated and compared using Fisher’s exact test. To account for multiple comparisons across outcomes, the Benjamini–Hochberg false discovery rate correction was applied to the interaction term *p* values from each mixed-effects model. A corrected *p* value less than 0.05 was considered to be statistically significant. Quantitative data were organized, processed and visualized using Microsoft Excel 2007 to identify significant trends and distributions.

## 3. Results

The baseline demographic, clinical, and laboratory characteristics of the study population are summarized in Table 1. A total of 14 pigs were included, with nine in the R group and five in the C group. Overall, baseline demographic, clinical, and laboratory characteristics were comparable between the R and C groups, with no statistically significant differences observed across all tested variables (*p* > 0.05).

### 3.1. Systemic Hemodynamic Measurements

**Figure 2:**
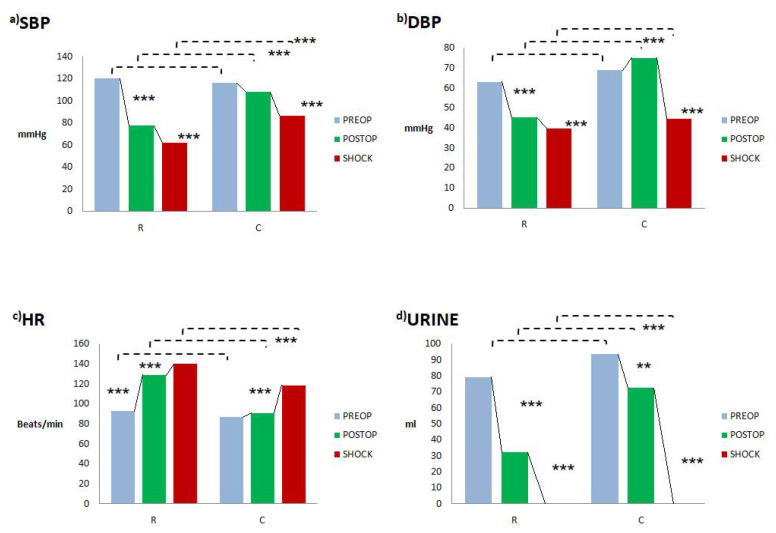
Hemodynamic parameters before and after RSD and during septic shock. (**a**) SBP: systolic blood pressure, (**b**) DBP: diastolic blood pressure, (**c**) HR: heart rate, (**d**) URINE: urine output. ** *p* < 0.01, *** *p* < 0.001.

Renal sympathetic denervation (RSD) induced a significant and sustained reduction in SBP and DBP within the R group. SBP decreased immediately postoperatively and remained reduced at 6 h after induction of septic shock (both *p* < 0.001, compared with the preoperative value), with significantly lower values in R than in C postoperatively (*p* < 0.001). The denervated group demonstrated a significantly earlier DBP drop, already evident at the time after laparotomy, surviving Benjamini–Hochberg correction (group × time interaction *p* BH < 0.001). Heart rate (HR) increased significantly within the R group postoperatively and 6 h later (both *p* < 0.001), with a significantly greater increase in the denervated group than in control (*p* BH = 0.0053). Urine output declined significantly in both groups, with an earlier and more-pronounced reduction in the R group at both postoperative timepoints (both *p* < 0.001, compared with the preoperative value), reaching statistical significance after correction between the two groups (*p* BH = 0.0107) (Figure 2).

### 3.2. Inflammatory Biomarkers

**Figure 3:**
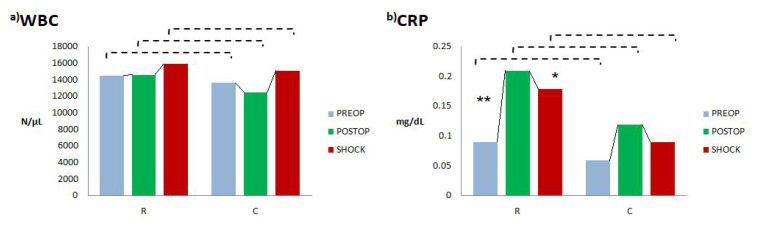
Inflammatory biomarkers before and after RSD and during septic shock. (**a**) WBC: white blood cell. (**b**) CRP: C-reactive protein. * *p* < 0.05, ** *p* < 0.01.

There were no significant changes in WBC within or between groups. CRP increased significantly in the R group postoperatively (*p* = 0.003) and at shock (*p* < 0.01). Although comparison between groups revealed a directional trend of significance with a *p* = 0.079, after correction with Benjamini–Hochberg, the effect was not confirmed at any timepoint (*p* BH = 0.3705) (Figure 3).

### 3.3. Coagulation System Markers

**Figure 4:**
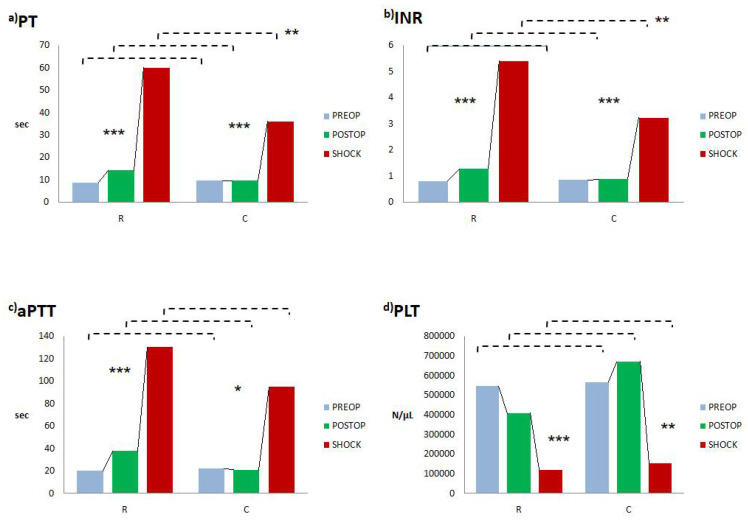
Coagulation system markers before and after RSD and during septic shock. (**a**) PT: prothrombin time, (**b**) INR: international normalized ratio, (**c**) aPTT: activated partial thromboplastin time, (**d**) PLT: platelet. * *p* < 0.05, ** *p* < 0.01, *** *p* < 0.001.

PT and INR did not change postoperatively but increased significantly at 6 h after induction of septic shock in the R group (*p* < 0.001), with a significantly greater increase than that in controls (*p* < 0.001 for both), also confirmed after correction (*p* BH = 0.0096). aPTT increased at shock within the R group (*p* < 0.0001) but did not differ from the control group after correction (*p* BH = 0.324). Platelet count decreased at 6 h in the R group (*p* < 0.001), without significant intergroup differences (Figure 4).

### 3.4. Renal Function and Electrolyte Biomarkers

BUN increased significantly at shock in the R group (*p* < 0.001), but changes were not significantly different from the control group after correction for multiple comparisons. CREA increased significantly at shock in both groups (*p*< 0.001 in R group and *p* = 0.039 in C group). Although initial comparison between groups showed significant difference with *p* = 0.04, it was not confirmed after Benjamini–Hochberg correction (*p* BH = 0.2171).

**Figure 5:**
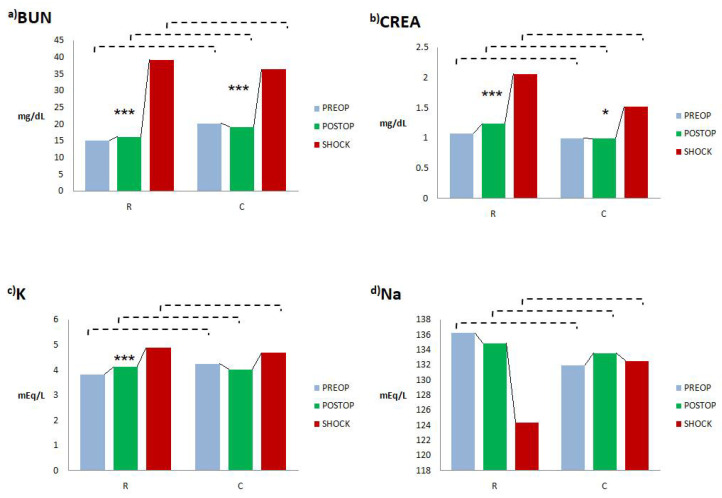
Renal function and electrolytes biomarkers before and after RSD and during septic shock. (**a**) BUN: blood urea nitrogen, (**b**) CREA: creatinine, (**c**) K: potassium, (**d**) Na: sodium. * *p* < 0.05, *** *p* < 0.001.

Potassium increased at shock within the R group (*p* < 0.001) without intergroup difference. Sodium remained unchanged and showed no meaningful trend in either group (Figure 5).

### 3.5. Hepatic Function Biomarkers

**Figure 6:**
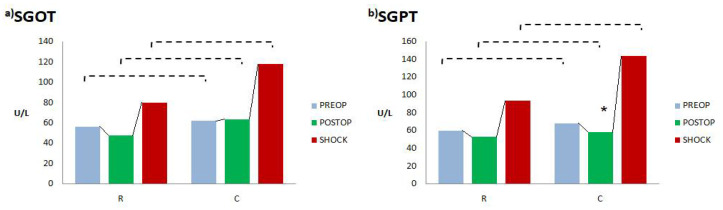
Hepatic function biomarkers before and after RSD and during septic shock. (**a**) SGOT: aspartate aminotransferase, (**b**) SGPT: alanine aminotransferase. * *p* < 0.05.

Hepatic function was based on SGOT and SGPT measurements as depicted in Figure 6. AST (SGOT) and ALT (SGPT) were not significantly affected within or between groups. SGPT showed a directional trend towards elevation after the shock induction (*p* = 0.078), but this was not confirmed after multiple corrections (*p* BH = 0.324).

### 3.6. Analysis of Alterations from Baseline to Septic Shock

Additionally, renal denervation’s effects on laboratory and clinical parameters were evaluated by comparing the change in values from preoperative to induction of shock between the R (denervation) and the C (control) groups (Table 2). Significant differences between groups were observed for SBP (*p* BH < 0.001), DBP (*p* BH < 0.001) HR (*p* BH = 0.005), URINE (*p* BH = 0.011), PT (*p* BH = 0.01) and INR (*p* BH = 0.01). All other parameters showed no significant intergroup differences over time (all *p* > 0.05).

### 3.7. Microscopic Findings and Histopathological Analysis

Table 3 represents the pathological lesions found microscopically in tissue sections after histopathological analysis. Multiorgan injury consistent with septic shock was observed. Although no lesion differed significantly between groups, directional trends were observed. Group R manifested lower hepatic degenerative changes, 22% in R vs. 80% in C (OR: 0.096, CI: 0.010, 4.143, *p* = 0.09); and higher pulmonary injury presented by atelectasis, 89% in R vs. 40% in C (OR: 9.034, CI: 0.679, 348.734, *p* = 0.094); and leukocytic infiltration, 78% in R vs. 20% in C (OR: 10.368, CI: 0.871, 387.909, *p* = 0.09). However, after Benjamini–Hochberg corrections, none of the above observed trends were confirmed (*p* BH = 0.5). Additionally, denervated animals manifested more-frequent glomerular damage and pulmonary alveolar damage, but this was not statistically different from the C group (both *p* = 0.265 and *p* BH = 0.687). Myocardial examination had no meaningful group separation.

## 4. Discussion

The main finding of this study is that bilateral renal sympathetic denervation (RSD) alters the hemodynamic and coagulation response during sepsis-induced shock in pigs, without considerably influencing renal failure progression, inflammatory response, or hepatic function. RSD significantly worsened hypotension (SBP dropped by 58 mmHg in the RSD group vs. 30 mmHg in controls, *p* < 0.001) and coagulopathy (PT prolonged by +51 s vs. +26 s, *p* < 0.001). No statistically significant differences were observed for renal dysfunction, inflammatory markers, or hepatic injury after correction for multiple comparisons. Directional trends were noted but did not reach significance. Only findings that survived Benjamini–Hochberg correction are interpreted with confidence; all remaining observations are reported as preliminary signals requiring confirmation in adequately powered studies. Moreover, the reported findings reflect the acute denervation context of this model and may not directly apply to patients with prior catheter-based RSD, in whom chronic adaptive changes would be expected to modify the physiological response.

Septic shock was successfully induced in both groups, as demonstrated by hypotension, tachycardia, decreased urine output, and significant increases in BUN and creatinine. Hyperkalemia occurred in denervated animals, while sodium levels and hepatic enzymes remained unchanged. These data are consistent with previous porcine studies describing hemodynamic instability and renal dysfunction in septic shock [24,32,33,34]. Given the close morphological and functional similarities between pigs and humans, notably regarding renal structure and response, this model offers translational relevance for understanding the acute physiological effects of renal sympathetic interruption during septic shock, though it does not replicate the chronic denervation context of patients with prior catheter-based RSD [35].

Coagulation disturbances were marked during shock. Across the study population, clotting times increased and platelet counts decreased, consistent with sepsis-associated coagulopathy secondary to endothelial injury, fibrinolysis and coagulation imbalance [36]. Notably, RSD was associated with significantly greater increases in PT and INR than those in the control group, and this difference survived Benjamini–Hochberg correction, making it the most robust findings of this study. This suggests a modulatory role of sympathetic signaling in coagulation dynamics. During septic shock, this procoagulation activity may partially counterbalance consumptive coagulopathy, and the interruption by RSD results in the rapid progress of coagulopathy, consistent with the greater prolongation of PT and INR observed in the study. The clinical significance of accelerated coagulopathy in denervated animals warrants attention, as coagulopathy is a major determinant of outcome in septic shock. However, direct extrapolation to patients with prior catheter-based RSD is not appropriate given the acute denervation context of this model, and dedicated studies are needed to fully characterize this relationship. PLT dropped over time in both groups but showed no significant group interaction, indicating that thrombocytopenia developed as a shared response to the sepsis model rather than as a denervation-specific phenomenon.

Inflammatory assessment showed a significant rise in CRP within the denervated group, whereas WBC remained unchanged. However, CRP changes were not significantly different from controls. As this difference did not survive correction for multiple comparisons, it should not be interpreted as a confirmed finding. However, whether RSD influences the inflammatory pathway in a longer observational window remains a question for future studies to answer, as WBC and CRP constitute nonspecific late markers, and the absence of cytokine measurements represents a critical gap that substantially limits the biological interpretation of the inflammatory findings. The absence of a confirmed inflammatory difference between groups does not constitute evidence against a neuroimmune modulatory role of renal sympathetic denervation.

Previous swine studies on RSD have reported reductions in arterial pressure and variable effects on renal perfusion, without consistent changes in renal function markers or electrolytes [23,37,38]. Some studies described histological vascular or parenchymal alterations after denervation, while others reported improved renal hemodynamics [22,39,40,41,42]; however, given the limited and conflicting nature of this evidence, direct comparisons with our findings should be made cautiously. Histological analysis in this study did not reveal statistically significant differences between groups. Nevertheless, the low statistical power of the study and the limited six-hour observation window may have missed capturing histological differences between groups.

The impact of bilateral sympathetic denervation of renal nerves in systemic response in a septic shock condition constitutes a subject that very few experimental studies have dealt with. Among these studies none used swine as experimental model, an animal more similar to the human organism by the prism of anatomy and architecture, urinary concentration, tolerance in tissue hypoxia and ischemia occurrence [35]. Experimental sepsis models in sheep and rodents similarly suggest that renal sympathetic nerves do not play a decisive role in the development of septic acute kidney injury [1,21,22].

In the present study, RSD was associated with a sustained reduction in systolic and diastolic blood pressure as well as a rapid increase in heart rate during septic shock, indicating a significant modulatory effect on hemodynamic regulation, with both findings confirmed after correction for multiple comparisons. It should be noted that the hemodynamic effects observed in this study were measured in the absence of vasopressor support. The interaction between renal sympathetic denervation and vasopressor therapy, particularly norepinephrine, warrants investigation in future studies, as sympathomimetic agents may partially compensate for or interact with the effects of denervation on vascular tone and renal perfusion [43]. Although BUN and creatinine increased in denervated animals, these changes did not differ significantly from controls, consistent with the view that septic renal failure progresses independently of renal sympathetic activity. Hepatic markers, electrolytes, and WBC showed no statistically confirmed intergroup differences after correction for multiple comparisons. Given the small sample size, limited statistical power, and short observation window, these negative findings should be interpreted as inconclusive rather than as evidence of no effect of RSD on these parameters.

Moreover, according to previous studies, organ dysfunction due to septic shock is depicted and confirmed by several histopathological changes after the microscopic evaluation of the tissue sections and histopathological analysis [24,44,45]. Histopathological analysis confirmed multiorgan injury consistent with septic shock. However, no statistically significant intergroup differences were observed in any tissue, and no histological finding survived Benjamini–Hochberg correction. Numerical differences in the frequency of pulmonary and hepatic lesions between groups are reported for descriptive completeness only and should not be interpreted as evidence of differential organ injury. Although hepatic degenerative changes did not reach statistical significance after correction for multiple comparisons, the observed difference between groups (22% in R vs. 80% in C, OR = 0.096, 95% CI: 0.003–1.148) represents a potentially clinically meaningful magnitude. This finding should be interpreted cautiously given the small sample size and low statistical power but warrants further investigation in adequately powered studies. Myocardial lesions were similar between groups. The limited observation window of six hours, while sufficient to capture hemodynamic and coagulation responses, likely precluded detection of fully developed histological organ injury, as sepsis-induced tissue changes are known to evolve progressively over longer timeframes [35,44,45].

Overall, renal sympathetic denervation in this porcine septic shock model exerts measurable effects on hemodynamic and coagulation parameters as well as urine output but does not produce confirmed alterations in renal dysfunction biomarkers, inflammatory response, or hepatic injury. These latter observations are specific to the acute denervation context and should not be directly extrapolated to patients who have previously undergone catheter-based RSD, in whom chronic adaptive changes may substantially alter the response to septic shock; these findings remain unconfirmed and require replication in larger, adequately powered studies before any conclusions can be drawn.

## 5. Conclusions

In this porcine model of fecal peritonitis-induced septic shock, bilateral renal sympathetic denervation altered cardiovascular and coagulation parameters, as evidenced by greater reductions in systolic and diastolic blood pressure, more-pronounced compensatory tachycardia, earlier decline in urine output, and greater prolongation of PT and INR than in controls, all confirmed after Benjamini–Hochberg correction. No statistically significant differences were detected between groups in renal dysfunction biomarkers, systemic inflammatory markers, hepatic injury parameters, or histopathological organ damage. These findings indicate a role for renal sympathetic signaling in circulatory and coagulation responses during septic shock, without evidence of harm from its interruption under the specific experimental conditions of this study.

Several limitations must be acknowledged. This study should be considered exploratory due to the small sample size and the large number of outcomes examined. The non-significant findings should be interpreted as inconclusive rather than as evidence of no effect, given the probability of type II error, as the study was underpowered for most endpoints. The six-hour observation window was sufficient for capturing acute hemodynamic and coagulation changes but likely insufficient for detecting the full evolution of histological organ injury, delayed inflammatory responses, and renal or hepatic dysfunction. The absence of direct denervation confirmation means incomplete denervation cannot be excluded and may have attenuated the observed effects. Inflammatory assessment was limited to nonspecific late markers poorly suited to detect differences in sympathetic modulation of inflammation. Future studies should be conducted examining cytokines, catecholamine and early organ injury biomarkers such as NGAL and KIM-1. The model represents untreated septic shock without vasopressor or antibiotic administration, limiting generalizability to current clinical practice. Furthermore, the acute denervation design of the study does not respond to the clinical scenario of patients prior to RSD who develop septic shock, as chronic denervation is associated with partial reinnervation and adrenergic receptor upregulation that may substantially modify the physiological response. Finally, hemodynamic monitoring relied on non-invasive sphygmomanometry without invasive arterial pressure monitoring, cardiac output measurement, or serum lactate quantification, and explicit predefined shock criteria were not established prospectively. Adequately powered studies incorporating chronic denervation models, invasive hemodynamic monitoring, serial lactate measurements, cytokine profiling, and standard resuscitative interventions are needed to clarify the full impact of renal sympathetic modulation in septic shock.

## Figures and Tables

**Table 1:** Baseline demographic, clinical and laboratory characteristics in total population and groups of study.

Characteristics	All (N = 14)	R Group (N = 9)	C Group (N = 5)	*p* Value
Sex (male/female)	10/4	6/3	4/1	0.266
Age (months)	13.7 ± 1.7	13.6 ± 1.7	14 ± 2	0.671
Weight (kg)	26.5 ± 1.9	26.1 ± 1.9	27.2 ± 1.9	0.326
SBP (mmHg)	118.9 ± 8.8	120.2 ± 8.2	116.4 ± 10.3	0.343
DBP (mmHg)	65.2 ± 6.4	63 ± 6.3	69 ± 4.9	0.1
HR (beats/min)	90.9 ± 8.5	93 ± 7.7	87.4 ± 9.7	0.398
URINE (mL)	84.6	79.4 ± 8.5	94 ± 19.2	0.097
WBC (N/μL)	14,241 ± 2656	14,511 ± 3142	13,680 ± 1620	0.637
CRP (mg/dL)	0.078 ± 0.07	0.087 ± 0.085	0.06 ± 0.025	0.561
PT (s)	9.31 ± 2.33	9.03 ± 2.31	9.82 ± 2.53	0.886
INR	0.83 ± 0.2	0.8 ± 0.2	0.88 ± 0.23	0.884
aPTT (s)	21 ± 24.9	20.2 ± 26.8	22.3 ± 24	0.921
PLT (N/μL)	554,571 ± 245,886	547,000 ± 210,829	568,200 ± 327,467	0.86
BUN (mg/dL)	17 ± 5.7	15.2 ± 4.5	20.2 ± 6.8	0.271
CREA (mg/dL)	1.05 ± 0.25	1.07 ± 0.27	1 ± 0.22	0.762
K (mEq/L)	4 ± 0.62	3.8 ± 0.7	4.2 ± 0.36	0.278
Na (mEq/L)	135 ± 6.7	136 ± 6.8	132 ± 6.2	0.711
SGOT (U/L)	58.6 ± 30.7	56.5 ± 31.5	62.2 ± 32.5	0.834
SGPT (U/L)	63.2 ± 19.9	60 ± 19	68.4 ± 22.6	0.772

SBP: systolic blood pressure, DBP: diastolic blood pressure, HR: heart rate, URINE: urine output, WBC: white blood cell, CRP: C-reactive protein, PT: prothrombin time, INR: international normalized ratio, aPTT: activated partial thromboplastin time, PLT: platelet count, BUN: blood urea nitrogen, CREA: creatinine, K: potassium, Na: sodium, SGOT: aspartate aminotransferase (AST), SGPT: alanine aminotransferase (ALT). Values represent mean ± SD. R group: renal denervation, C group: control.

**Table 2:** Changes from baseline to septic shock.

	Group	Alteration After Shock	95% CI	*p* Value After Shock	95% CI	*p* Value R vs. C	*p* Interaction BH
SBP (mmHg)	R	−57.67	(−65.12, −50.22)	<0.001	(16.16, 32.33)	<0.001	<0.001
	C	−29.6	(−39.6, −19.6)	<0.001			
DBP (mmHg)	R	−23.33	(−29.73, −16.93)	<0.001	(−2.26, 12.31)	0.17	<0.001
	C	−30.2	(−32.79, −15.61)	<0.001			
HR (beats/min)	R	+47.56	(35.16, 59.95)	<0.01	(−34.47, −8.41)	<0.01	0.0053
	C	+31.6	(14.97. 48.23)	<0.01			
URINE (mL)	R	−81.67	(−94.54, −68.79)	<0.001	(−14.72, 14.72)	1	0.0107
	C	−94	(−111.28, −76.72)	<0.001			
WBC (N/μL)	R	+1463	(−2007.11, 4933.78)	0.647	(−4377.43, 2708.54)	0.636	0.549
	C	+1460	(−3196.09, 6116.09)	0.921			
CRP (mg/dL)	R	+0.092	(0.01, 0.17)	<0.01	(−0.18, 0.01)	0.079	0.3705
	C	+0.034	(−0.07, 0.14)	0.448			
PT (s)	R	+51.13	(40.05, 62.22)	<0.001	(−35.23, −12.98)	<0.001	0.0096
	C	+26.24	(11.37, 41.11)	<0.001			
INR	R	+4.61	(3.62, 5.6)	<0.001	(−3.17, −1.17)	<0.001	0.0096
	C	+2.37	(1.04, 3.7)	<0.001			
aPTT (s)	R	+110.68	(67.11, 154.24)	<0.001	(−79.41, 8.03)	0.107	0.324
	C	+72.86	(14.41, 131.31)	0.013			
PLT (N/μL)	R	−427,444	(−649,544.23, −205,344.66)	<0.001	(−211,434.49, 278,323.38)	0.783	0.2187
	C	−415,200	(−713,178.12, −117,221.88)	<0.01			
BUN (mg/dL)	R	+24.11	(17.21, 31.01)	<0.001	(−11.83, 6.36)	0.543	0.2187
	C	+16.4	(7.15, 25.65)	<0.001			
CREA (mg/dL)	R	+0.993	(0.61, 1.37)	<0.001	(−1.06, −0.02)	0.04	0.2171
	C	+0.532	(0.02, 1.04)	0.04			
K (mEq/L)	R	+1.06	(0.47, 1.64)	<0.001	(−0.93, 0.53)	0.581	0.2187
	C	+0.46	(−0.32, 1.24)	0.342			
Na (mEq/L)	R	−11.89	(−35.34, 11.56)	0.474	(−15.38, 31.69)	0.487	0.4821
	C	+0.6	(−30.86, 32.06)	1			
SGOT (U/L)	R	+23.78	(−30.03, 77.59)	0.602	(−16.18, 92.71)	0.163	0.4514
	C	+56.40	(−15.79, 128.59)	0.148			
SGPT (U/L)	R	+33.33	(−17.11, 83.78)	0.254	(−6.05, 106.71)	0.078	0.324
	C	+75.60	(7.93, 143.28)	0.0267			

SBP: systolic blood pressure, DBP: diastolic blood pressure, HR: heart rate, URINE: urine output, WBC: white blood cell, CRP: C-reactive protein, PT: prothrombin time, INR: international normalized ratio, aPTT: partial thromboplastin time, PLT: platelet count, BUN: blood urea nitrogen, CREA: creatinine, K: potassium, Na: sodium, SGOT: aspartate aminotransferase (AST), SGPT: alanine aminotransferase (ALT). Values are mean ± SD. R group: renal denervation, C group: control.

**Table 3:** Histopathological analysis.

Tissue	Pathological Lesion	Groups		OR	95% CI	*p* Value	*p* Interaction BH
		**R**	**C**				
Kidney	Acute Tubular Injury	78%	80%	0.920	(0.024, 15.096)	1	1
	Glomerular Damage	33%	80%	0.157	(0.004, 1.762)	0.265	0.687
	Interstitial Capillary Congestion	100%	100%	NC	NC	1	1
	Microhemorrhages	78%	60%	2.176	(0.162, 30.473)	0.58	0.844
Lung	Alveolar Damage	78%	40%	4.468	(0.412, 66.083)	0.265	0.687
	Edema	33%	40%	0.761	(0.069, 9.531)	1	1
	Leukocytic Infiltration	78%	20%	10.368	(0.871, 387.909)	0.09	0.5
	Capillary Congestion	100%	100%	NC	NC	1	1
	Atelectasis	89%	40%	9.034	(0.679, 348.734)	0.094	0.5
	Microhemorrhages	56%	20%	4.185	(0.375, 146.356)	0.3	0.687
Heart	Edema—Interfibrillar Distance	78%	80%	0.920	(0.024, 15.096)	1	1
	Capillary Congestion	100%	80%	NC	NC	0.357	0.714
	Microscopic Bleeding	44%	60%	0.566	(0.046, 5.636)	1	1
	Leukocytic Infiltration	33%	20%	1.807	(0.143, 65.053)	1	1
Liver	Capillary Congestion	78%	100%	NC	NC	0.5	0.844
	Degenerative Changes	22%	80%	0.096	(0.003, 1.148)	0.09	0.5
	Portal Tract Inflammation	56%	80%	0.359	(0.010, 4.143)	0.58	0.844
	Kupffer Cell Hyperplasia	67%	100%	NC	NC	0.258	0.687

## Data Availability

The data presented in this study are available on request from the corresponding author.

## Abbreviations

The following abbreviations are used in this manuscript:

SNS	Sympathetic Nervous System
RSD	Renal Sympathetic Denervation
SBP	Systolic Blood Pressure
DBP	Diastolic Blood Pressure
HR	Heart Rate
WBC	White Blood Cell Count
CRP	C-Reactive Protein
PT	Prothrombin Time
INR	International Normalized Ratio
aPTT	Activated Partial Thromboplastin Time
PLT	Platelet Count
BUN	Blood Urea Nitrogen
CREA	Creatinine
AST/SGOT	Aspartate Aminotransferase
ALT/SGPT	Alanine Aminotransferase
K	Potassium
Na	Sodium
OR	Odds Ratio
ER	Expected Frequency
BH	Benjamini–Hochberg
